# Sounds from the Eocene: the first singing cicada from the Messel Pit, Germany

**DOI:** 10.1038/s41598-025-94099-7

**Published:** 2025-04-29

**Authors:** Hui Jiang, Maxwell S. Moulds, Stephan M. Blank, Jes Rust, Sonja Wedmann

**Affiliations:** 1 Senckenberg Forschungsstation Grube Messel, Senckenberg Forschungsinstitut und Naturmuseum Frankfurt/Main, Markstraße 35, 64409 Messel, Germany; 2 Institute of Geology and Paleontology, Charles University, Albertov 6, Prague 2, Prague, 12843 Czech Republic; 3 Abteilung Paläontologie, Bonner Institut für Organismische Biologie (BIOB), Universität Bonn, Nußallee 8, 53115 Bonn, Germany; 4 State Key Laboratory of Paleobiology and Stratigraphy, Nanjing Institute of Geology and Palaeontology, Chinese Academy of Sciences, Nanjing, 210008 China; 5 Australian Museum Research Institute, Sydney, NSW 2010 Australia; 6 Senckenberg Deutsches Entomologisches Institut, Eberswalder Straße 90, 15374 Müncheberg, Germany

**Keywords:** Palaeontology, Taxonomy, Biogeography, Macroecology

## Abstract

Cicadidae is one of the most species-rich insect families today. However, compared to the number of extant species, fossil records of Cicadidae are extremely limited. Among singing cicadas, the tribe Platypleurini within the Cicadinae subfamily is notable for its broad geographic distribution, high species diversity, and distinctive features, but no reliable fossil records have been discovered to date. In this study, we report the first fossil record of the Platypleurini from the Eocene Messel Pit: a new genus and species, *Eoplatypleura messelensis*. This species not only represents one of the earliest known fossil crown-group Cicadidae from the Eurasian continent but also the oldest confirmed record of the subfamily Cicadinae worldwide to date. As the first described fossil singing cicada from the Eocene Messel Pit, this discovery enriches the species diversity of the Messel biota and fills a significant gap in the Eocene cicada fossil record. The discovery of *E. messelensis* gen. et sp. nov. will serve as a fossil calibration point for future molecular phylogenetic studies and provides new insights into the origins and dispersal patterns of Platypleurini. Based on the analysis of existing records, Cicadidae was once widely distributed in Germany and across Europe during the Cenozoic.

## Introduction

Cicadidae (cicadas) are well-known for the evolution of their extensive sound production systems, exceptional long-term juvenile subterranean habits, and symbolic attributes and utility across various cultural, life, and scientific studies e.g.,^1–6^. Cicadidae belong to the superfamily Cicadoidea, which consists of two extant families: Cicadidae and Tettigarctidae^2,7^ . Extant Cicadidae are widely distributed across all continents except Antarctica, with approximately 450 genera and more than 3000 species currently documented^8,9^. In contrast, extant Tettigarctidae comprises only one genus with two species, both of which are endemic to Australia^10–12^. The earliest fossil records of Cicadoidea, dating back to the upper Triassic in Australia and South Korea, were classified within Tettigarctidae^13,14^. However, recent research by Jiang et al. (2024)^6^ demonstrates that many Mesozoic fossils previously attributed to Tettigarctidae might actually belong to stem cicadids, suggesting that further examination of early Mesozoic fossils is necessary to understand the phylogenetic relationships between fossils and modern groups and their evolutionary history better.

**Fig. 1:**
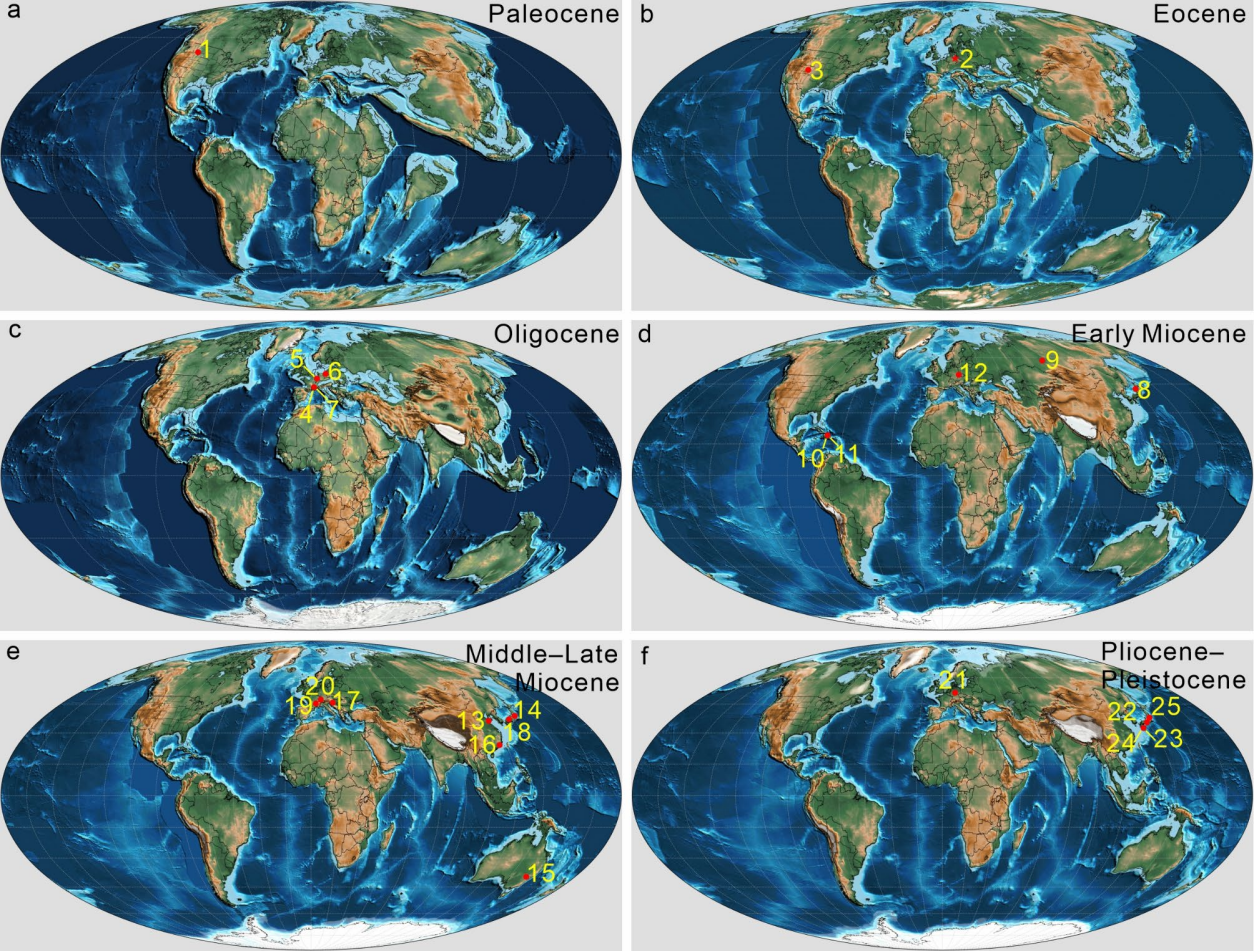
Paleogeographic map of Cicadidae fossil distribution. The numbers on the map represent different fossil sites. Numbers: (1) Bear Creek, Montana, USA; (2) Messel, Germany; (3) Florissant, USA; (4) Céreste, France; (5) Kleinkems, Germany; (6) Seifhennersdorf, Germany; (7) Aix-en-Provence, France; (8) Sado, Japan; (9) W Siberia, Russia; (10) Cordillera Septentrional, Dominican Republic; (11) La Búcara, Dominican Republic; (12) Bílina, Czech Republic; (13) Shanwang, China; (14) Nasu Volcano, Japan; (15) New South Wales, Australia; (16) Zhangpu, China; (17) Radoboj, Croatia; (18) Tottori Pref., Japan; (19) Andance, France; (20) Oeningen, Switzerland; (21) Willershausen, Germany; (22) Hyogo Pref., Japan; (23) Kashiyama, Japan; (24) Kagoshima Pref., Japan; (25) Shiobara, Japan. For specific species information from each site, see Table 1. Map source: Scotese et al., 2024^15^. Refer to the literature on fossil localities in a checklist of fossil records of Cicadidae in Table 1.

The previously hypothesized earliest fossil of Cicadidae, initially believed to be a first-instar nymph from the mid-Cretaceous Kachin amber of Myanmar and the only Mesozoic record, was reevaluated by Jiang et al. (2024)^6^. After analysing additional nymphal fossils from Kachin amber and comparing them with modern Cicadoidea nymphs, they found the evidence insufficient to categorize this fossil within the crown group of Cicadidae, suggesting instead its affiliation with a stem group. To date, 44 fossil records of Cicadidae from 24 Cenozoic sites have been documented (Table 1; Fig. 1). The earliest known fossil record of an adult Cicadidae is a forewing discovered in the Late Paleocene deposits near Bear Creek, Montana, USA^16^ (Table 1; Fig. 1). Molecular evidence, based on estimated origin times for various Hemipteran groups, suggests that Cicadidae originated between 160 Ma and 40 Ma^17,18^. A key factor contributing to this wide range of time of origin is the limited selection of modern taxa, scarce use of dated fossils, and issues with the taxonomic classification of fossils. The precise origination times of Cicadidae currently are inconclusive. Our updated checklist of Cicadidae fossil records (Table 1) reveals that, aside from the earliest confirmed adult Cicadidae fossil from the Paleocene, subsequent records are primarily from the Oligocene and later periods. Notably, there is an apparent gap of cicada fossils in the Eocene—the interval between the Paleocene and Oligocene. This suggests a considerable gap in our knowledge, emphasizing the need to review older literature and search for fossil collections, both of which warrant further investigation.

**Table 1:** A checklist of fossil records of Cicadidae. Tribal and generic placements follow Moulds (2018)^19^ where they differ from those originally published.

No.	Age (Ma)	Time interval	Species	Tribe	No.	Locality	Reference
1	59.2–56.0	Late Paleocene	*Davispia bearcreekensis* Cooper, 1941	Tibicinini	1	Bear Creek, Montana, USA	Cooper, 1941^16^
2	48–47	Middle Eocene	*Eoplatypleura messelensis* gen. et sp. nov.	Platypleurini	2	Messel	This work
3	34	Late Eocene	*Hadoa grandiosa* Scudder, 1892	Cryptotympanini	3	Florissant, Colorado, USA	Scudder, 1892^20^; Moulds, 2018^19^
4	34	Late Eocene	*Lithocicada perita* Cockerell, 1906	Tibicinini		Florissant, Colorado, USA	Cockerell, 1906^21^
5	34	Late Eocene	*Platypedia primigenia* Cockerell, 1908	Platypediini		Florissant, Colorado, USA	Cockerell, 1908^22^
6	33.9–27.8	Early Oligocene	*Paracicadetta oligocenica* Boulard & Nel, 1990	Pagiphorini	4	Céreste, France	Boulard & Nel, 1990^23^
7		Early Oligocene	Cicadidae gen. et sp. indet.		5	Kleinkems, Germany	Théobald, 1937^24^
8	30.5–30.2	Early Oligocene	*Lyristes* sp.	Cryptotymp anini	6	Seifhennersdorf, Germany	Tietz et al., 1998^25^; Moulds, 2018^19^
9	27.8–26.0	Late Oligocene	*Cicadatra? serresi* Meunier, 1915	?Cicadatrini	7	Aix-en-provence, France	Meunier, 1915^26^; Moulds, 2018^19^
10	24	Early Miocene	Cicadidae gen. et sp. indet.		8	Sado, Japan	Takahashi et al., 2024^27^
11	23–16	Early Miocene	*Tymocicada gorbunovi* Becker-Migdisova, 1954	Dundubiini	9	W Siberia, Russia	Becker-Migdisova, 1954^28^; Moulds, 2018^19^
12	20–15	Early/Middle Miocene	*Dominicicada youngi* Poinar & Kritsky, 2011	Cicadinae	10	Cordillera, Septentrional, Dominican Republic	Poinar and Kritsky, 2012^29^
13	20–15	Early/Middle Miocene	*Minyscapheus dominieanus* Poinar, Kritsky & Brown, 2011	Taphurini	11	La Búcara, Dominican Republic	Poinar et al., 2012^30^
14	17.9–17.8	Early Miocene	*Tibicina sakalai* Prokop & Boulard, 2000	Tibicinini	12	Bílina, Czech Republic	Prokop and Boulard, 2000^31^
15	16.0–11.6	Middle Miocene	*Cryptotympana incasa* Zhang, Sun & Zhang, 1994	Cryptotympanini	13	Shanwang, Shandong, China	Zhang et al., 1994^32^; Moulds, 2018^19^
16	16.0–11.6	Middle Miocene	*Cryptotympana miocenica* Zhang & Zhang,1990	Cryptotympanini		Shanwang, Shandong, China	Zhang and Zhang; 1990^33^; Moulds, 2018^19^
17	16.0–11.6	Middle Miocene	*Tanyocicada lapidescens* Zhang, 1989	Leptopsaltriini		Shanwang, Shandong, China	Zhang, 1989^34^; Moulds, 2020^35^
18	16-11.6	Middle Miocene	*Meimuna protopalifera* Fujiyama, 1969	Dundubiini	14	Nasu Volcano, Japan	Fujiyama, 1969^36^
19	16.0–11.6	Middle Miocene	*Laopsaltria ferruginosa* Moulds, Frese & McCurry, 2022	Cicadettini	15	New South Wales, Australia	Moulds et al., 2022^37^
20	16.0–11.6	Middle Miocene	*Burbungoides gulgongensis* Moulds, Frese & McCurry, 2022	Burbungini		New South Wales, Australia	Moulds et al., 2022^37^
21	16.0–11.6	Middle Miocene	*Tithopsaltria titan* Moulds, Frese & McCurry, 2022	Arenopsaltriini		New South Wales, Australia	Moulds et al., 2022^37^
22	14.7	Middle Miocene	Cicadidea gen. et sp. indet.		16	Zhangpu, China	Wang et al., 2021^38^
23	13.8–11.6	Middle Miocene	*Camuracicada aichhorni* Heer, 1853	Cryptotympanini	17	Radoboj, Croatia	Heer, 1853^39^; Moulds, 2018^19^
24	13.8–11.6	Middle Miocene	*Paleopsalta ungeri* Heer, 1853	Cicadettini		Radoboj, Croatia	Heer, 1853^39^; Moulds, 2018^19^
25	11.6–5.3	Late Miocene	*Auritibicen* sp. aff. *japonicus* Kato, 1925	Cryptotympanini	18	Tottori Pref., Japan	Kinugasa and Miyatake, 1976^40^; Moulds, 2018^19^
26	11.6–5.3	Late Miocene	*Graptopsaltria inaba* Fujiyama, 1982	Polyneurini		Tottori Pref., Japan	Fujiyama, 1982^41^
27	11.6–5.3	Late Miocene	*Yezoterpnosia* sp. aff. *vacua* Olivier, 1790	Leptopsaltriini		Tottori Pref., Japan	Kinugasa and Miyatake, 1979^42^; Moulds, 2018^19^
28	8.5-8.0	Late Miocene	*Lyristes renei* Riou, 1995	Cryptotympanini	19	Andance, France	Riou, 1995^43^
29	8.5–8.0	Late Miocene	*Tibicina gigantea* Boulard & Riou, 1988	Tibicinini		Andance, France	Boulard and Riou, 1988^44^
30	8.5–8.0	Late Miocene	*Miocenoprasia grasseti* Boulard & Riou, 1999	Lamotialnini		Andance, France	Boulard and Riou, 1999^45^
31	7.2–5.3	Late Miocene	*Lyristes? emathion* Heer, 1853	Cryptotympanini	20	Oeningen, Switzerland	Heer, 1853^39^
32	3.6–2.6	Late Pliocene	*Cicada* aff. *orni* Linnaeus, 1758	Cicadini	21	Willershausen, Germany	Wanger, 1967^46^; Moulds, 2018^19^; Moulds et al. 2023^47^
33	3.6–2.6	Late Pliocene	*Cicada* aff. *lodosi* Boulard, 1979	Cicadini		Willershausen, Germany	Moulds et al. 2023^47^
34	3.6–2.6	Late Pliocene	*Cicada tithonus* Moulds, Kaulfuss & Gehler, 2023	Cicadini		Willershausen, Germany	Moulds et al. 2023^47^
35	3.6–2.6	Late Pliocene	*Tibicina* aff. *haematodes* Scopoli, 1763	Tibicinini		Willershausen, Germany	Moulds, 2018^19^; Moulds et al. 2023^47^
36	3.6–2.6	Late Pliocene	*Tibicina lata* Moulds, Kaulfuss & Gehler,2023	Tibicinini		Willershausen, Germany	Moulds et al. 2023^47^
37	3.6–2.6	Late Pliocene	*Tibicina* sp.	Tibicinini		Willershausen, Germany	Moulds et al. 2023^47^
38	3.6–2.6	Late Pliocene	*Tibicina boulardi* Moulds, Kaulfuss & Gehler,2023	Tibicinini		Willershausen, Germany	Moulds et al. 2023^47^
39	2.6–2.3	Early Pleistocene	*Meimuna* sp.	Dundubiini	22	Hyôgo Pref., Japan	Fujiyama, 1982^41^
40	2.6–2.3	Early Pleistocene	*Graptopsaltria* sp.	Polyneurini		Hyogo Pref., Japan	Fujiyama, 1982^41^
41	2.6–2.3	Early Pleistocene	*Euterpnosia* sp.	Leptopsaltriini		Hyogo Pref., Japan	Inoue,1986^48^
42	1.7–1.5	Early Pleistocene	*Tanna?* sp.	Leptopsaltriini	23	Kashiyama, Japan	Fujiyama, 1979^49^
43	1.1–0.5	Middle Pleistocene	*Graptopsaltria* aff. *nigrofuscata* Motschulsky, 1866	Polyneurini	24	Kagoshima Pref., Japan	Fujiyama, 1979^49^
44	0.038–0.033	Late Pleistocene	*Auritibicen bihamatus* Motschulsky, 1861	Cryptotympanini	25	Shiobara, Tochigi Pref., Japan	Fujiyama, 1979^49^; Moulds, 2018^19^
45	0.038–0.033	Late Pleistocene	*Yezoterpnosia nigricosta* Motschulsky, 1866	Leptopsaltriini		Shiobara, Tochigi Pref., Japan	Fujiyama, 1969^49^; Moulds, 2018^19^

**Fig. 2:**
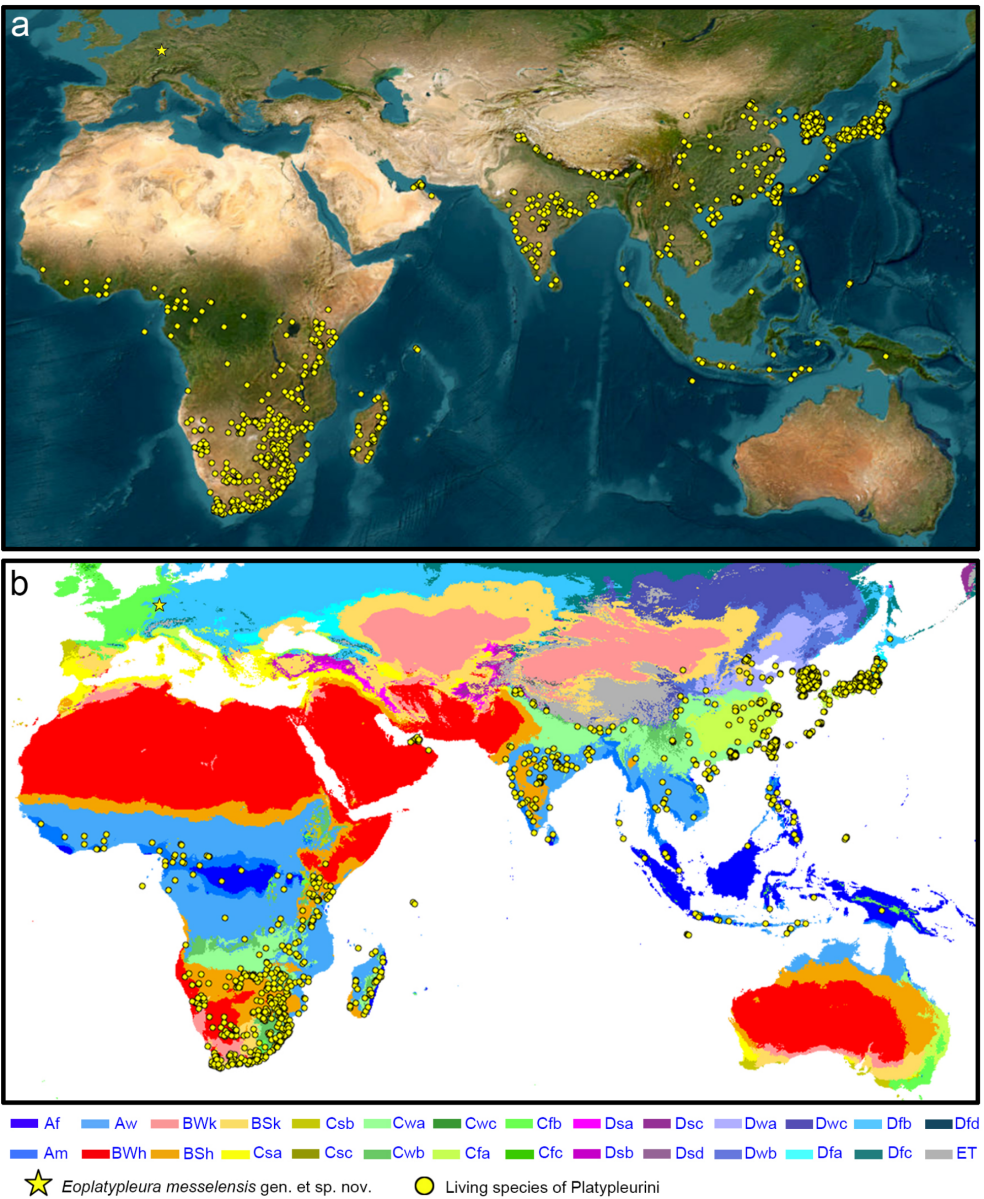
Global distribution maps of Platypleurini. (**a**) Showing Platypleurini distribution on satellite map. Map source: Esri, Maxar, Earthstar Geographics, and the GIS User Community (acrgis pro 3.0.0; https://www.arcgis.com/home/item.html?id=10df2279f9684e4a9f6a7f08febac2a9). (**b**) Showing Platypleurini distribution on Köppen-Geiger climate classification map (1980–2016). Map source: Beck et al., 2018^51^. The data of living species from GIBF (https://www.gbif.org/) and iNaturalist (https://www.inaturalist.org/). Climate classification system of (**b**) based on the Köppen climate classification. *Af* Tropical rainforest climate, *Am* Tropical monsoon climate, *BWh* Hot desert climate, *BWk* Cold desert climate, *BSh* Hot semi-arid climate, *BSk* Cold semi-arid climate, *Cfa* Humid subtropical climate, *Cfb* Temperate oceanic climate, *Cfc* Subpolar oceanic climate, *Cwa* Monsoon, *Cwb* Subtropical highland climate, *Cwc* Cold subtropical highland climate, *Csa* Hot-summer Mediterranean climate, *Csb* Warm-summer Mediterranean climate, *Csc* Cold-summer; Mediterranean climate, *Dfa* Hot-summer humid continental climate, *Dfb* Warm-summer humid continental climate, *Dfc* Subarctic climate, *Dwa* Monsoon-influenced hot-summer humid continental climate, *Dwb* Monsoon-influenced warm-summer humid continental climate, *Dwc* Monsoon-influenced subarctic climate, *Dwd* Monsoon-influenced extremely cold subarctic climate, *Dsa* Mediterranean-influenced hot-summer humid continental climate, *Dsb* Mediterranean-influenced warm-summer humid continental climate, *Dsc* Mediterranean-influenced subarctic climate, *Dsd* Mediterranean-influenced extremely cold subarctic climate, *ET* Tundra climate.

In this study, we describe the first species of a singing cicada from the Eocene Messel, Germany. The two specimens are assigned to the tribe Platypleurini based on the characteristics of its preserved wings and body. Platypleurini is a highly remarkable group within the Cicadidae, currently comprising about 36 extant genera (see in Results section), primarily distributed in tropical and subtropical regions of sub-Saharan Africa and Asia (Fig. 2). Molecular studies and biogeographical analyses suggest that this tribe originated in Africa, with a solitary dispersal event occurring after the collision of Africa and Eurasia (about 30–25 Ma, Oligocene)^50^. They propose that this event may have facilitated the colonization that led to the establishment of the clade of Asian platypleurines. Previously, only a fossil identified as *Platypleura *sp. from the Early Oligocene of Kleinkems (Germany, Baden-Württemberg), was attributed to the genus *Platypleura*^24^. However, upon re-examination of a photo of this specimen, we found that the specimen retains general Cicadidae morphology, including an identifiable head and operculum in ventral view, but lacks well-preserved wings or other specific taxonomic features. Consequently, this renders its preservation quality inadequate for classification within the genus *Platypleura* or the tribe Platypleurini. The here described new species is notable for being the earliest known fossil record of the family Cicadidae on the Eurasian continent and the earliest global record of the Cicadinae subfamily. As the first described singing cicada fossil from the Eocene Messel fossil pit, this specimen offers new insights into the origins and dispersal of Platypleurini, enriches the species diversity of the Messel pit, and fills a gap in the true cicada palaeontological record of the Eocene.

## Materials and methods

Two fossil cicada specimens from the Messel Pit, Messel, near Darmstadt in Hesse, Germany were studied. They are deposited in the “collection of invertebrates from the Messel Pit fossil site” of the Senckenberg Forschungsinstitut und Naturmuseum Frankfurt/M. (SF), which is located at the Senckenberg Forschungsstation Grube Messel. The specimens of extant *Planopleura kaempferi* were collected by H. Sauter from Taihanroku, the southernmost port of Formosa (Hengchun Township, Taiwan, R.O.C.; Fig. 7h) and deposited at Senckenberg Deutsches Entomologisches Institut, Müncheberg, Germany and one was collected in Nanjing, Jiangsu Province, China (Fig. 7i).

The two fossil specimens were photographed using a Leica M165 C microscope with a JENOPTIK GRYPHAX microscope camera. Figure 7h was photographed using a Nikon D7200 camera with an AF-S VR Micro-NIKKOR 105 mm f/2.8G ED lens and a Yongnuo YN-560-TX Flash Controller. Composite images were compiled with Zerene Stacker. Scanning electron microscopic (SEM) investigation of *Platypleura kaempferi* was performed after coating the specimen with gold. Photos were taken with a Tescan LYRA3 microscope at 15 kV acceleration voltage. Single SEM images were stitched in Adobe Photoshop CS6. Digital overlay drawings were created using Procreate and an Apple iPad Air tablet. Figures were prepared in Adobe Photoshop CS6 and CorelDraw 2021. The global distribution of Platypleurini on the satellite map and Köppen-Geiger climate classification maps (1980–2016) were made in ArcGIS Pro. The source of the satellite map is from Esri, Maxar, Earthstar Geographics, and the GIS User Community. The source of satellite map Köppen-Geiger climate classification map (1980–2016) is from Beck et al., 2018^51^ based on the Köppen climate classification. The distribution data of living species are taken from GIBF (https://www.gbif.org/) and iNaturalist (https://www.inaturalist.org/). The artistic reconstruction in Fig. 11 was created using Pixologic ZBrush, Maya, and Adobe Photoshop CC.

Wing venation terminology follows Moulds, 2005^2^, 2012^3^. Figure 7h,i depict *Planopleura kaempferi* from the Platypleurini tribe, which can be compared with the fossil members of the same tribe shown in Fig. 7d,e to identify the basal structures and details around the node. Figure 8e,f illustrate the extant hindwings of *Plan. kaempferi* and *Yanga guttulata*, used for comparison with fossil hindwings.

### Geological and paleoenvironmental setting of Grube Messel

**Fig. 3:**
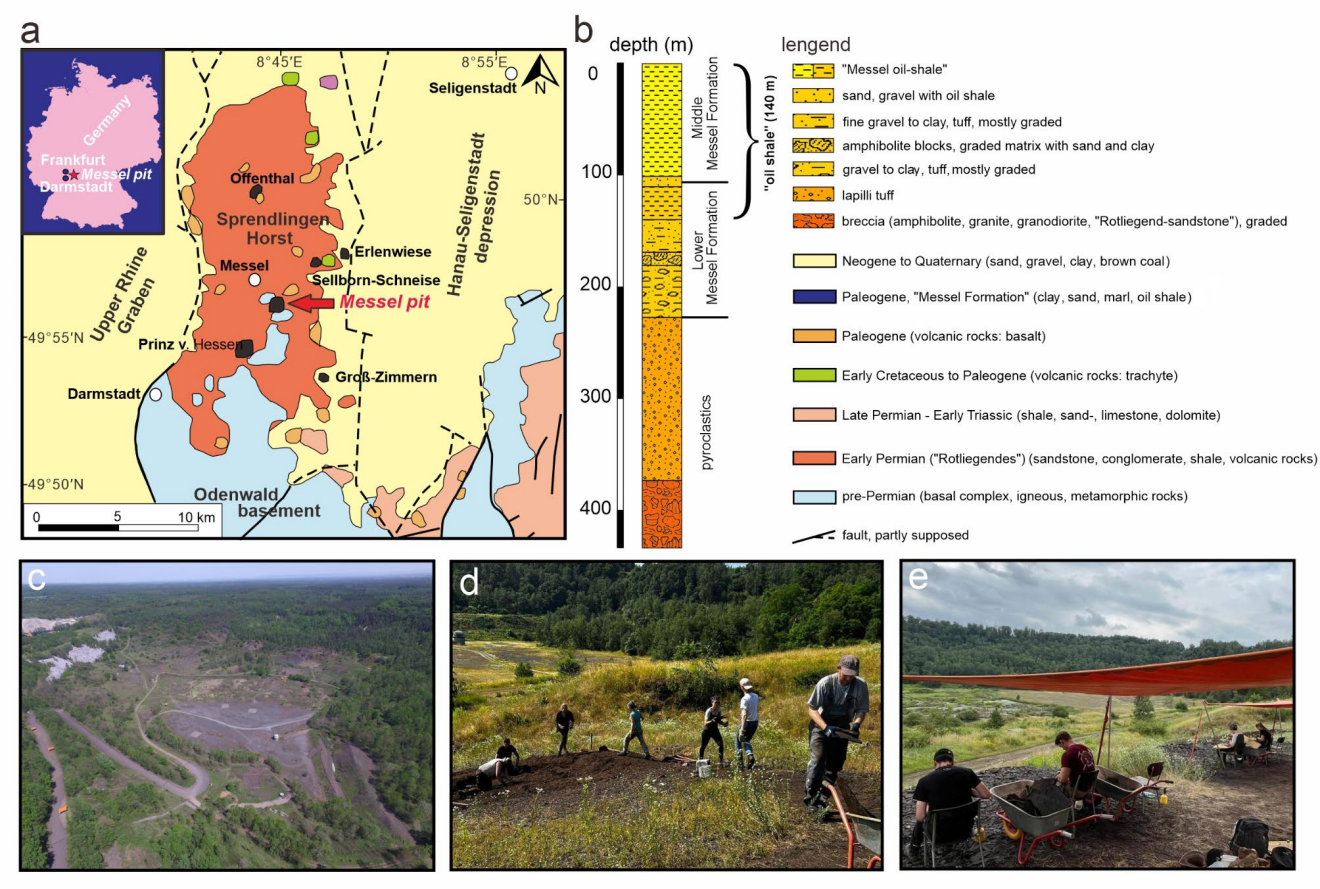
(**a**) Geological map showing the location of the Messel pit in relation to other Paleogene sites on the Palaeozoic basement of the Sprendlinger Horst (modified after^53^, based^54^). (**b**) Generalised section of the Messel 2001 core (modified after^53^) displaying the major lithological units. (**c**) Aerial panoramic view of the Messel Pit, photo taken by Sven Tränkner. (**d**) Photo of fossil collection on-site, taken by Nicolas Adrian Stagg. (**e**) Photo of fossil processing on-site, taken by Loup Boudet.

The Messel Pit, once an oil shale open-cast mine, lies 10 km northeast of Darmstadt in the German state of Hesse (Fig. 3a). It is located on the Sprendlinger Horst, an uplifted block of Palaeozoic basement^54,56,57^. The Messel formation was deposited in a maar lake formed by phreatomagmatic eruptions which was confirmed in the year 2001 by a 433 m long research drill core^55^ (Fig. 3b). The drillcore encompasses the entire sequence of lacustrine sediments from the so-called Lower Messel Formation (LMF) and approximately 60% of the Middle Messel Formation (MMF). Utilizing astronomical tuning, it has been determined that the LMF and MMF sediments cover a c. 840 kyr interval between 48.06 Ma and 47.22 Ma^58^. The fossils are embedded in organic-rich fine-grained sediments, commonly called oil shale^59^. Mean annual temperatures (MAT) have been estimated using plant macrofossils and microfossils through a coexistence approach (CA) at the family level, along with leaf margin analysis (LMA). The MAT ranged from 16.8 to 23.9 °C based on CA, and from 21.7 to 23.1 °C based on LMA, with an overall average MAT of approximately 22 °C^60^. The coldest month temperatures exceeded 10 °C, supported by the presence of thermophilic reptiles such as crocodiles^61,62^. Mean annual precipitation may have reached a maximum of about 2540 mm and relative humidity of 75 ± 2% was also inferred using the CA method^60^. Figure 3c–e shows an aerial panoramic view of the Messel Pit, along with the fossil collection and processing on-site.

## Results

### Systematic classification

Superfamily Cicadoidea Batsch, 1789^63^.

Family Cicadidae, Batsch, 1789.

Subfamily Cicadinae, Batsch, 1789.

Tribe Platypleurini Schmidt, 1918^64^.

Type genus. *Platypleura* Amyot & Serville (type species *Cicada stridula* L.).

Included genera (updated after^2,7^): *Afzeliada* Boulard, 1972^65^; *Albanycada* Villet, 1989^66^; *Attenuella* Boulard, 1972^65^; *Azanicada* Villet, 1989^66^; *Brevisiana* Boulard, 1972^65^; *Canualna* Boulard, 1985^67^; *Capcicada* Villet, 1989^66^; *Eopycna* Sanborn, 2020^68^; *Esada* Boulard, 1972^65^; *Hamza* Distant, 1904a^69^; ?*Hainanosemia* Kato, 1927^70^; *Ioba* Distant, 1904b^71^; ?*Kalabita* Moulton, 1923^72^; *Karscheliana* Boulard, 1990^23^; *Koma* Distant, 1904b^71^; *Kongota* Distant, 1904b^71^; *Muansa* Distant, 1904b^71^; *Munza* Distant, 1904b^71^; *Neoplatypleura* Kato, 1926^73,74^; *Orapa* Distant, 1905^75^; *Oxypleura* Amyot & Audinet-Serville, 1843^76^; *Planopleura* Lee, 2024^73^; *Platypleura* Amyot & Audinet-Serville, 1843^76^; *Pycna* Amyot & Audinet-Serville, 1843^76^; *Pycnoides* Sanborn, 2020^68^; *Sadaka* Distant, 1904b^71^; *Sechellalna* Boulard, 2010^77^; *Severiana* Boulard, 1972^65^; *Soudaniella* Boulard, 1972^65^; *Strumosella* Boulard, 1972^65^; *Strumoseura* Villet, 1999^78^; *Suisha* Kato, 1928^79^; *Tigripleura* Lee, 2024^73^; *Ugada* Distant, 1904b^71^; *Umjaba* Distant, 1904b^71^; *Yanga* Distant, 1904b^71^. “?” indicates uncertainty regarding the taxonomic placement of the genus.

### Genus *Eoplatypleura* gen. nov.

#### Zoobank LSID: urn:lsid:zoobank.org:act:D2BD5436-1FCF-4E4B-AE51-44038D6F34AF

##### Type species

*Eoplatypleura messelensis* sp. nov.; by present designation.

##### Etymology

The new genus-group name combines the word “Eocene” with the tribe name “Platypleurini ”, as current identifications indicate that this fossil species is most closely related in morphology to the Platypleurini. The genus name is also based on the ancient Greek noun ή πλευρά (ē pleura), which is feminine in grammatical gender.

##### Diagnosis

Head width, including compound eyes, slightly narrower than anterior margin of pronotum; supra-antennal plate separated from eyes at a distance; forewings with eight apical cells; length of forewing apical cells 3 and 4 close to one third of forewing length; forewing broad, length/width ratio ~ 2.4; forewing veins C and Sc widely separated and costal margin strongly ampliated and strongly curved in proximal part; veins M and CuA relatively wide apart at basal cell, vein M more distant than CuA; fused section of vein M about equal in length to following veins of M_1 + 2_ and M_3 + 4_ intersected with nodal line, and slightly shorter than half length of vein CuA; CuA_1_ intersected by crossvein m-cu at about mid length; wings with coloration.

**Fig. 4:**
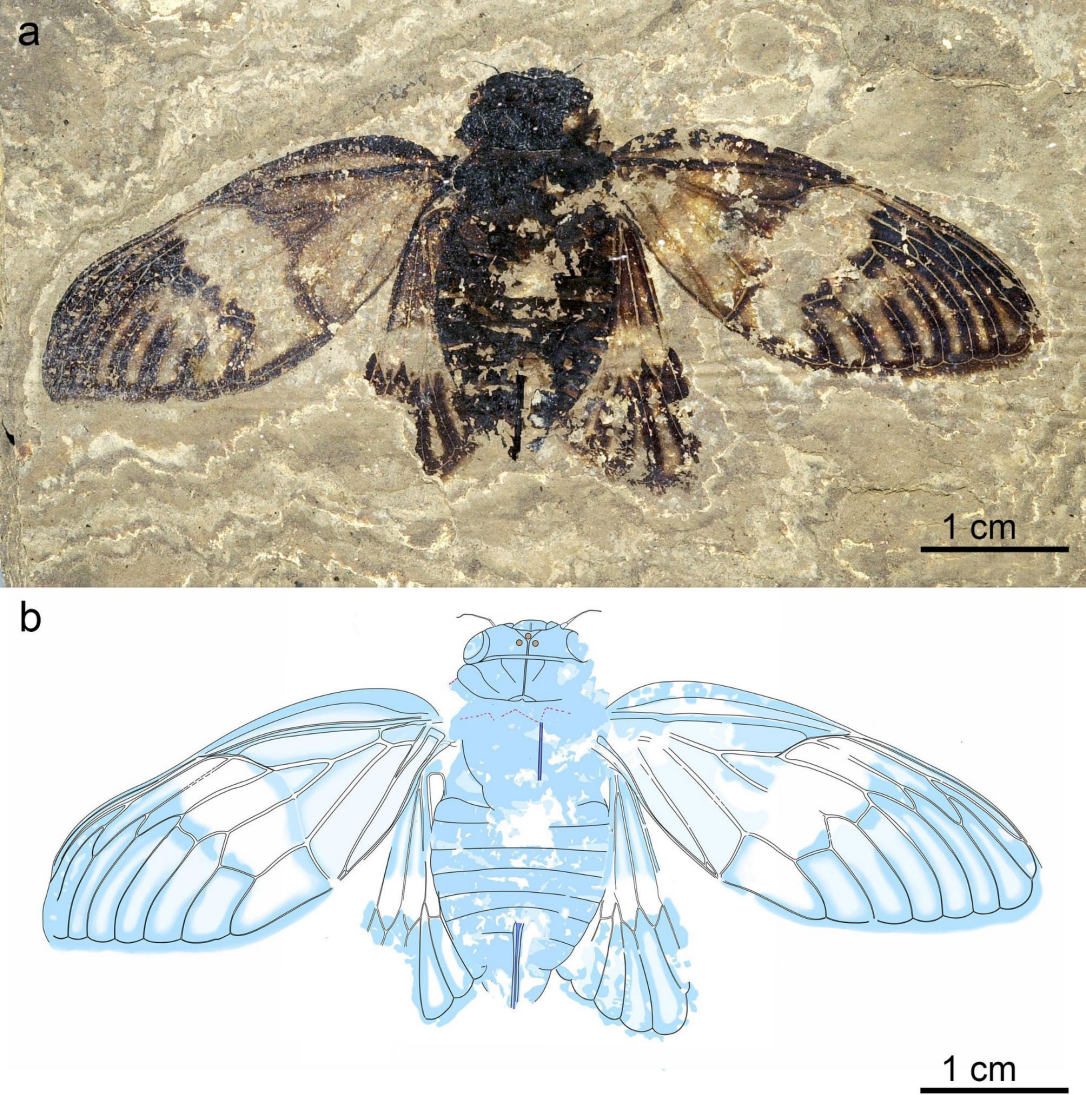
*Eoplatypleura messelensis* gen. et sp. nov., holotype, SF-Mel1515. (**a**) Photographic overview. (**b**) Overlay drawing overview.

**Fig. 5:**
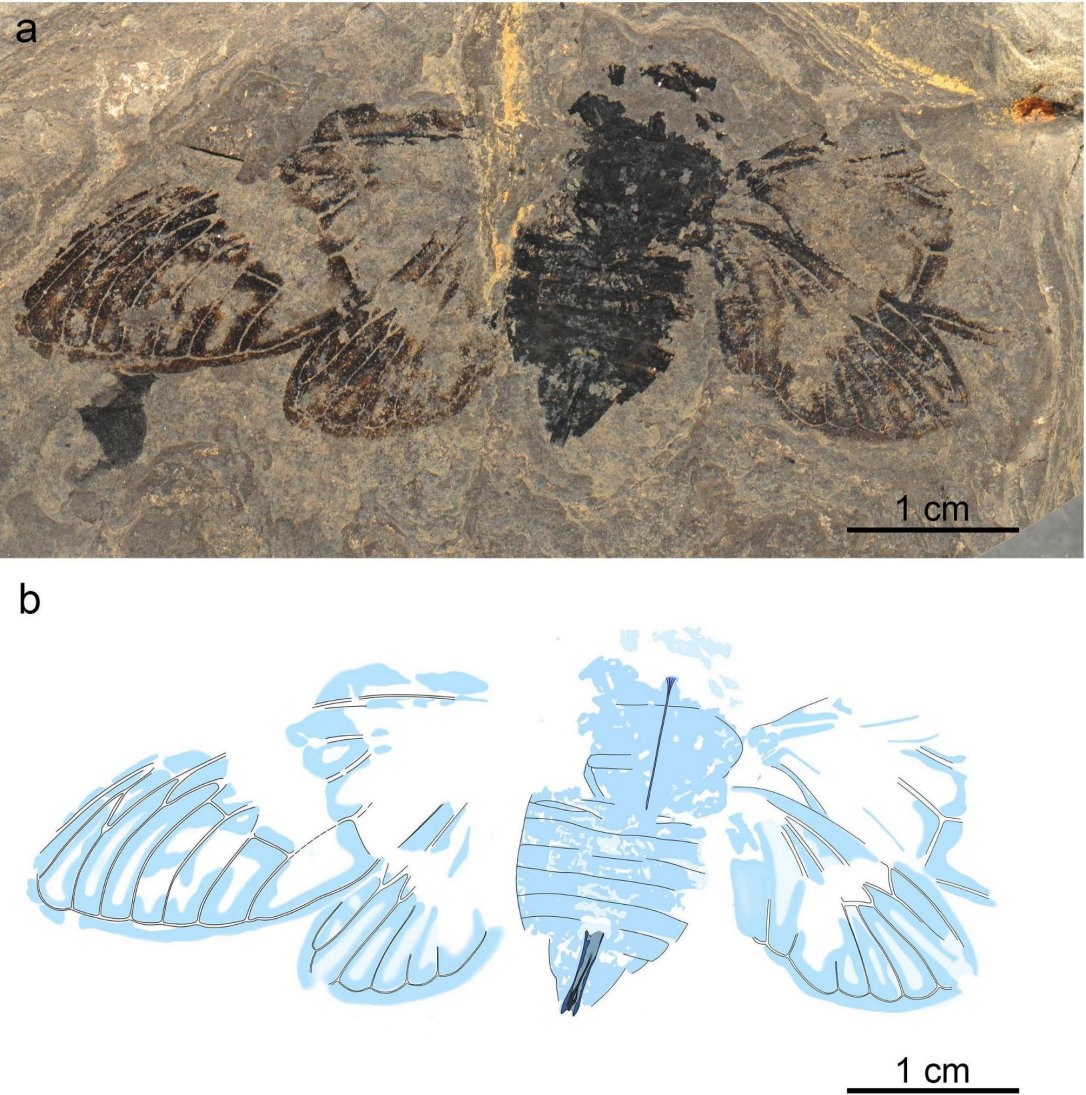
*Eoplatypleura messelensis* gen. et sp. nov., paratype, SF-Mel8954. (**a**) Photographic overview. (**b**) Overlay drawing overview.

### *Eoplatypleura messelensis* sp. nov.

#### Zoobank LSID: urn:lsid:zoobank.org:act:39E425C0-C683-4294-9E36-6C6EA4AA0BF8 

**Fig. 6:**
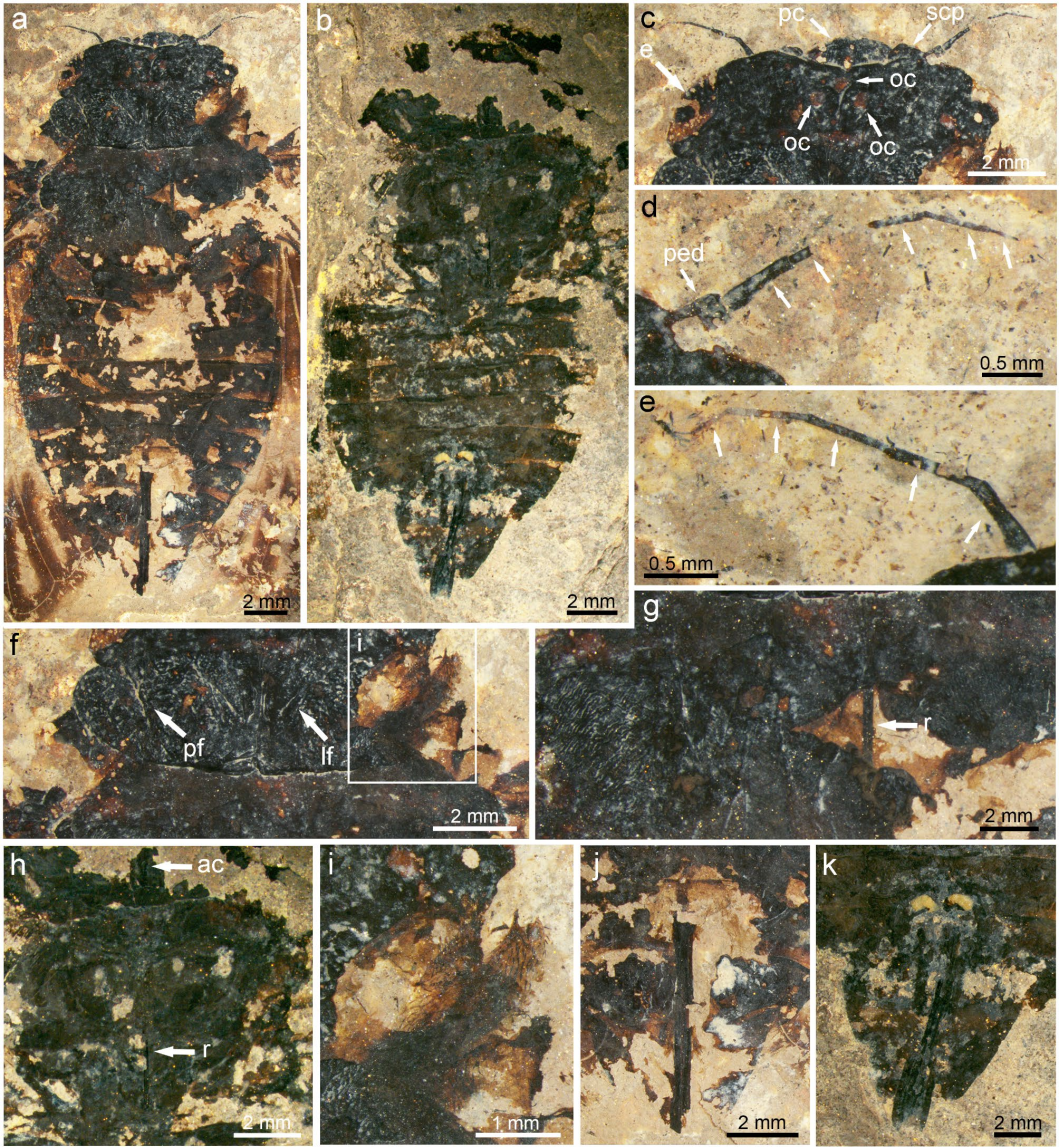
The body structures of *Eoplatypleura messelensis* gen. et sp. nov. excluding the wings. (**a**), (**c**–**g**), (**i**), (**j**) Holotype, SF-Mel1515. (**b**), (**h**), (**k**) Paratype, SF-Mel8954. (**a**) Dorsal view of the non-winged body of holotype. (**b**) Ventral view of the non-winged body of holotype. (**c**) Head in dorsal view of holotype. (**d**) Right antenna in detail of paratype. (**e**) Left antenna in detail of holotype. (**f**) Pronotum of holotype. (**g**) Mesonotum of holotype, showing fine surface wrinkling ornamentation, with broken areas revealing the rostrum. (**h**) Thorax in ventral view of paratype. (**i**) Part of the leg covered in hairs of holotype. (**j**) Ovipositor of holotype. (**k**) Ovipositor of paratype. Abbreviations:* ac* anteclypeus;* e* compound eye;* lf* lateral fissure;* pc* postclypeus;* ped* pedicel;* pf* paramedian fissure;* r* rostrum;* scp* scape; and* oc* ocellus.

**Fig. 7:**
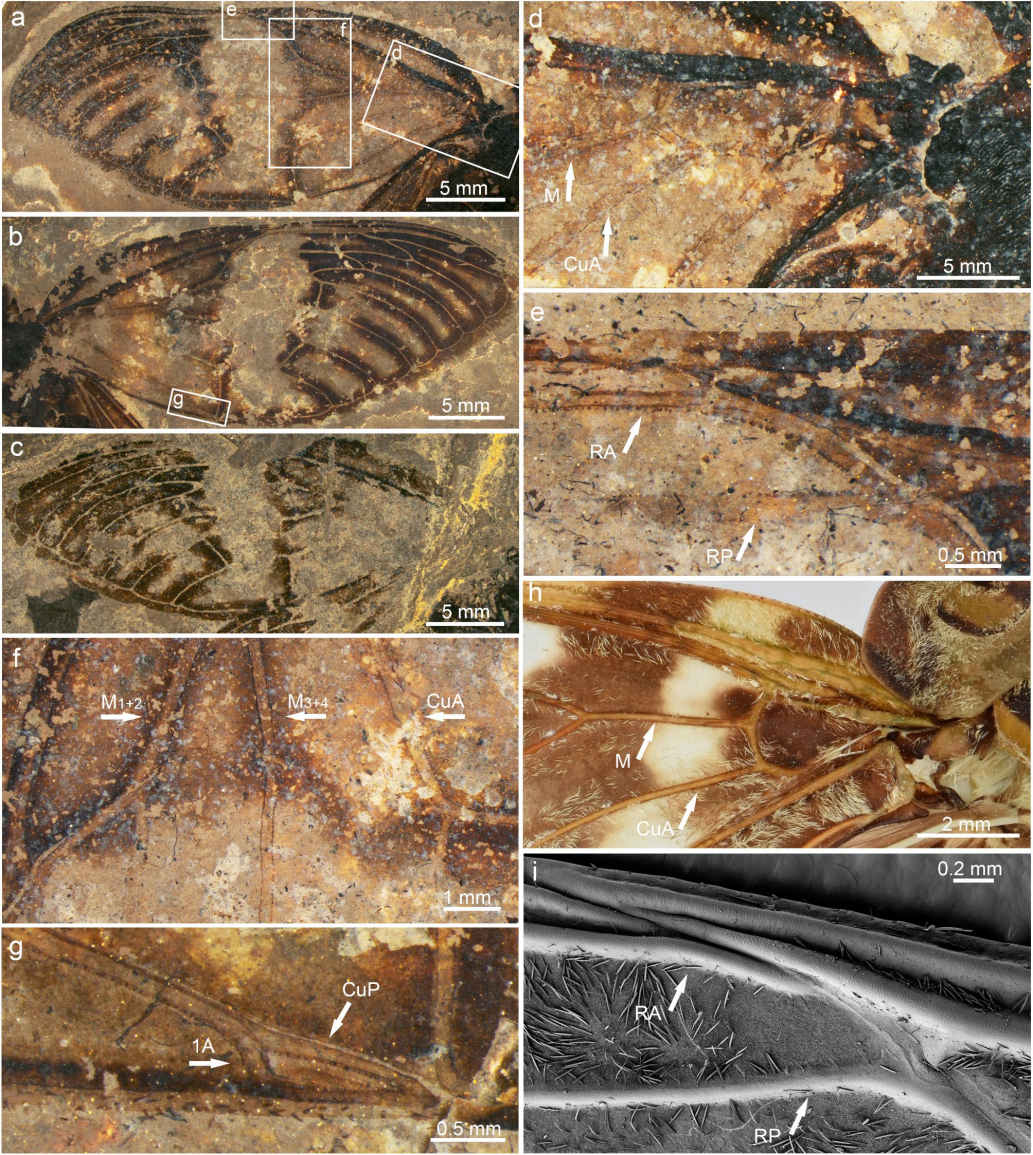
Forewing structures of *Eoplatypleura messelensis* gen. et sp. nov. and living species. (**a**), (**b**) and (**d**–**f**) Holotype SF-Mel1515; (**c**) and (**g**) Paratype SF-Me18054. (**a**) Holotype left forewing. (**b**) Holotype right forewing. (**c**) Paratype right forewing. (**d**) Showing basal part from (**a**). (**e**) holotype showing details of the area around node from (**a**). (**f**) Showing middle part of holotype forewing from (**a**). (**g**) Showing detail of terminals of CuP and 1 A from (**c**). (**h**) Showing details of the basal part of forewing of *Planopleura kaempferi*. (**i**) Showing details of the area around node of *P. kaempferi*, SEM image.

**Fig. 8:**
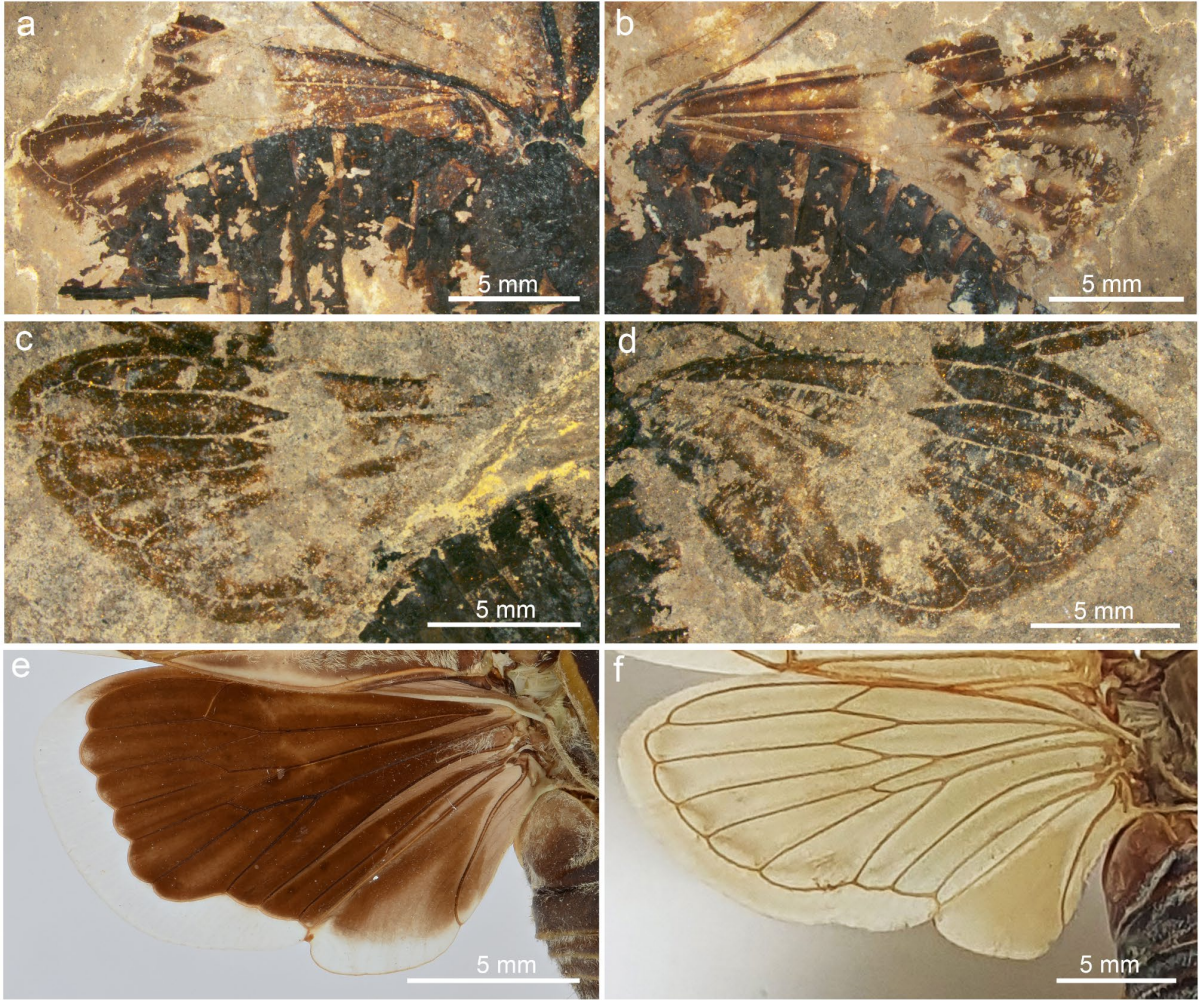
Hindwing structures of *Eoplatypleura messelensis* gen. et sp. nov. and living species. (**a**) and (**b**) Holotype SF-Mel1515. (**a**) left hindwing. (**b**) right hindwing. (**c**) and (**d**) Paratype SF-Mel8954. (**c**) right hindwing. (**d**) left hindwing. (**e**) left hindwing of *Planopleura kaempferi*. (**f**) right hindwing of *Yanga guttulata*.

**Fig. 9:**
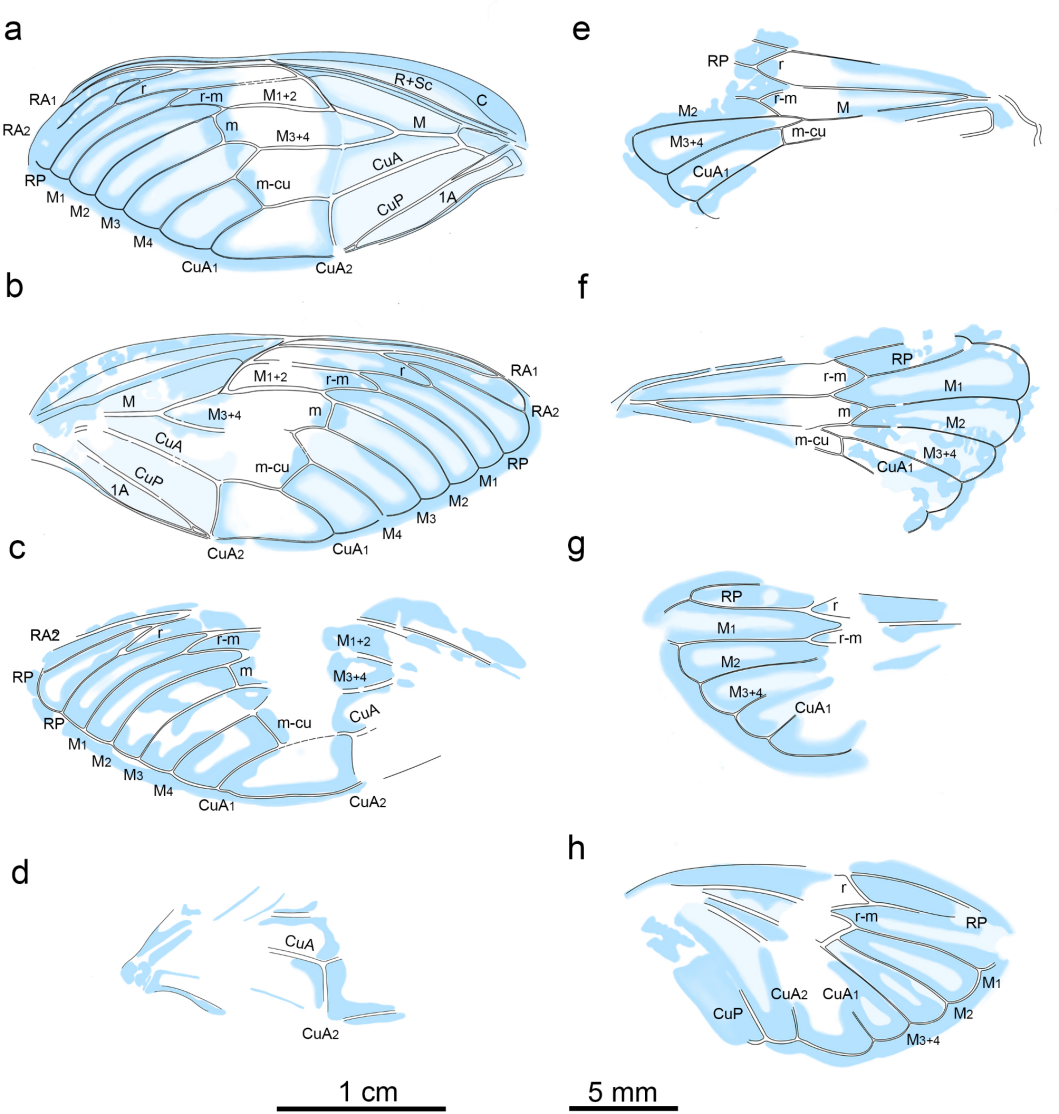
Overlay drawing of wing structures of *Eoplatypleura messelensis* gen. et sp. nov. (**a**), (**b**), (**e**) and (**f**) Holotype SF-Mel1515. (**c**), (**d**), (**g**) and (**h**) Paratype SF-Mel8954. (**a**) holotype left forewing. (**b**) holotype right forewing. (**c**) paratype right forewing. (**d**) paratype left forewing. (**e**) holotype left hingwing. (**f**) holotype right hindwing. (**g**) paratype right hindwing. (**h**) paratype left hindwing. Scale bar: (**a**–**d**), 1 cm; (**e**–**h**), 5 mm.

(Figs. 4, 5, 6, 7, 8 and 9).

##### Etymology

The species name attributes to the fossil pit of Messel. The species name is an adjective in genitive singular.

##### Material

Holotype, coll. no. SF-Mel1515, adult female, relatively completely preserved in dorsal view, in spread-wing condition, pronotal collar not clearly preserved (Fig. 4). Paratype, coll. no. SF-Mel8954, adult female, preserved in ventral view, in spread-wing condition, head and most of right forewing missing, part of right hind femur and tibia visible (Fig. 5). Body of holotype ca. 26.5 mm long, ca. 12.2 mm wide, wingspan ca. 68.2 mm. Body of paratype ca. 23.1 mm long, ca. 10.7 mm wide, wingspan ca. 58.7 mm.

##### Type locality and horizon

Grube Messel (latitude 49°55’N, longitude 8°45’E), Hesse, Germany; Messel Formation, Lutetian, Eocene; Holotype SF-Mel1515 was collected in 1986 at digging site 29 in grid square H12/13 in strata around local stratigraphic marker horizon M. Based on the study of Kaboth-Bahr et al. (2024)^58^ the sediment layer around marker horizon M can be estimated to have an age of c. 47.2 ± 0.21 Ma. Paratype SF-Mel8954 was collected in the Messel pit in the 1980ies by Christa Behnke and donated to Senckenberg in 1999, precise data are not known.

##### Diagnosis

As for the genus due to monotypy. Additional characteristics include wings with extensive coloration; dark bands along veins, and a hyaline patch in the middle; the margin membranes of the wings are distinct and coloured. The widest width of the forewing margin membrane is far narrower than half the width of apical cell 8 (Fig. 9a–c).

Description: Head (Fig. 6a–e). Width of head including compound eyes slightly narrower than width of anterior margin of pronotum. Longest diameter of compound eye in dorsal view shorter than half distance between compound eyes; vertex with three rounded, well-developed ocelli. Postclypeus bulging in ventral view, ca. 2.3 mm long, ca. 0.7 mm wide. Supra-antennal ledge visible, separated from eyes at a distance; five left and six right antennomeres visible in dorsal view; pedicel of right antenna visible, ca. 2.1 times as long as wide; flagella slender. Rostrum of holotype only partially preserved and visible through decayed thorax; rostrum of paratype preserved, reaching first abdominal segment.

Thorax (Fig. 6f–h). Pronotum of holotype ca. 2.8 mm long, ca. 9.2 mm wide (except pronotal collar); pronotal lateral and ambient fissures developed, forming distinct eminences in central and lateral area; mesonotum exposed but incomplete, slightly broader than pronotum (except collar), ca. 10.7 mm wide, some fine wrinkles visible; distal structure of mesonotum unpreserved.

Opercula unknown.

Wings (Figs. 7, 8 and 9). Forewing (Fig. 7a–g). Left forewing of holotype about 29.4 mm long, about 12.6 mm wide; distal upper part slightly bent downwards; right forewing about 30.2 mm long, 12.6 mm wide. Left forewing of paratype about 29.4 mm long, about 12.1 mm wide; right forewing only preserved in a small portion of anterior part. Forewings of both holotype and paratype with extensive coloration, dark bands along veins and nodal line, hyaline patch in middle, membranous margins broad and with dark coloration. Node at of about 0.49 wing length. RP separated from vein Sc + R at about 0.42 wing length. Sc separated from vein Sc + RA at about 0.48 wing length, terminating at node. RA divided into veins RA_1_ and RA_2_ at about 0.67 wing length; RP and M_1_ connected by crossvein r-m at about 0.66 wing length. M separated from basal cell at about 0.14 wing length, and then divided into veins M_1 + 2_ and M_3 + 4_ at about 0.26 wing length; M_1 + 2_ divided into veins M_1_ and M_2_ at about 0.59 wing length; M_2_ and M_3_ connected by crossvein m at about 0.61 wing length; M_3 + 4_ divided into veins M_3_ and M_4_ at about 0.54 wing length. CuA divided into CuA_1_ and CuA_2_ at about 0.38 wing length; M_4_ and CuA_1_ connected by crossvein m-cu at about 0.54 wing length; CuA_2_ terminates at nodal clave about 0.37 wing length; veins CuP and 1 A fuse for much of their length, beginning at about 0.15 of wing length, and diverging near nodal clavus, close to their terminus; CuP terminates at nodal clave. Nodal line traceable linking RA + Sc, RP, M_1 + 2_, M_3 + 4_, and CuA. Marginal membrane very broad.

Hindwing (Fig. 8a–d). Hindwings incompletely preserved, wings of holotype folded along body sides; hindwings of paratype spread out. Hindwings with fused stem of RP and M short but well formed; with six apical cells; apical cell 1 more than half length of apical cell 2; apical cell 2 long; apical cells 3–5 long and similar; extensive coloration, dark bands along veins, hyaline patch in middle; membranous margins broad and with dark coloration.

Legs. Dense hairs present on foreleg remains of holotype (Fig. 6i).

Abdomen. Abdomen slightly broader than thorax, abdomen of holotype about 15.3 mm long, about 12.1 mm wide; abdomen of paratype about 12.5 mm long, about 10.8 mm wide; sternites II to VIII with narrowed light brown band on posterior margin.

Genitalia. Ovipositor not extending beyond abdominal apex (Fig. 6j,k).

## Discussion

### The taxonomic placement of *Eoplatypleura messelensis* gen. et sp. nov.

The newly discovered *E. messelensis* gen. et sp. nov., which conforms to the tribe Platypleurini, exhibits the following characteristics: the head is not produced, with its width across the eyes being nearly equal to the width of anterior margin of the pronotum (Fig. 6a); the compound eyes are not overly protruding (Fig. 6c); the length of the pronotum (except the pronotal collar) is narrow (Fig. 6a,f); the forewing is broad (Fig. 7a–c); the costal margin of the forewings is prominently ampliated and arched at the base; forewings and hind wings feature variegated coloration (Figs. 4 and 5). This combination of characters makes *E. messelensis* gen. et sp. nov. morphologically compatible to the tribe Platypleurini within the Cicadidae.

The following combined characters are sufficient to distinguish *E. messelensis* gen. et sp. nov. from all known genera within the tribe Platypleurini: the forewing is broad but with a more subelliptical outline; the lateral anterior part of the pronotal collar is not laterally expanded when viewed dorsally; the margin membranes of the wings display coloration; and the widest width of the forewing margin membrane is narrower than half the width of the cell. Some features are not common among other groups in the Cicadidae family. For example, the broad forewing with a strongly ampliated and strongly curved costal margin at the base is more similar to genera such as *Yanga* and *Pycna* from Madagascar, which belong to the first clade branching off in the Platypleurini tribe, with the origin of lineage extending back to the middle Oligocene as suggested by Price et al. (2019)^50^. The pronotal collar with a strongly ampliated lateral margin is a common characteristic of the Platypleurini. However, in *E. messelensis* gen. et sp. nov., the lateral anterior pronotal collar is not laterally expanded when viewed dorsally. This variation may represent either a retained ancestral trait or a derived characteristic of *E. messelensis* gen. et sp. nov., indicating a potential deviation from the crown group of Platypleurini and necessitating further fossil specimens to confirm its significance.

Wing coloration patterns are characteristic of many species within the tribe Platypleurini. These patterns are commonly believed to serve a camouflage role, such as when the platypleurines perch on tree trunks^66,80^. *E. messelensis* gen. et sp. nov. features an extensive wing colour pattern. This pattern may have a similar function to those in modern platypleurines, which are adapted to forest or woodland environments, especially considering the subtropical forest environment of Messel during the period 48–47 Ma.

Currently, no morphological phylogenetic analyses have been conducted for the Platypleurini, and the features preserved in *E. messelensis* gen. et sp. nov. are relatively limited compared to modern species, making it challenging to determine its relationships within the Platypleurini groups based solely on morphology. Price et al. (2019)^50^ conducted a molecular phylogenetic study of the Platypleurini, providing significant insights into their internal relationships, and Marshall et al. (2018)^7^ provided a comprehensive molecular phylogeny of the Cicadidae, including the phylogenetic relationship between Platypleurini and its sister groups. Based on the phylogenetic relationships inferred via molecular analyses, it can be inferred that *E. messelensis* gen. et sp. nov. may be classified into one of three most likely and more specific taxonomic positions. Possibility one: *E. messelensis* gen. et sp. nov. may be classified as a stem group within the Platypleurini tribe. Given its Eocene provenance and the result that it clearly predates the molecular clock estimates for the origin of the Platypleurini ^50^, along with possessing characteristics that distinguish it from the extant Platypleurini discussed above, *E. messelensis* gen. et sp. nov. likely represents one of the most basal members of the Platypleurini. Possibility two: *E. messelensis* gen. et sp. nov. might be classified as a member associated with the African forest lineage of the Platypleurini tribe. Given that the environmental conditions of the Messel forest bear a resemblance to those of the African forest habitats, and the morphology of *E. messelensis* gen. et sp. nov. shows notable similarities to the genera *Yanga* and *Pycna* found in Madagascar, it is possible that *E. messelensis* gen. et sp. nov. could belong to this lineage. Possibility three: *E. messelensis* gen. et sp. nov. might be classified as a member associated with the extant Asian lineage of the Platypleurini tribe. *E. messelensis* gen. et sp. nov. was discovered in Europe, and fossil evidence indicates that the Cicadidae were widely distributed across Eurasia during the Cenozoic (Fig. 1). Current data do not rule out the possibility that the Platypleurini may have originated early in Eurasia, or that *E. messelensis* gen. et sp. nov. could have derived from an early dispersion across the Eurasian continent. Thus, the potential that *E. messelensis* gen. et sp. nov. stems from this lineage remains open.

### Implications for the origin and divergence timing of the tribe platypleurini

The discovery of *E. messelensis* gen. et sp. nov. fills a gap in the fossil record of the family Cicadidae from the Eocene. It represents not only the earliest and sole fossil record of the tribe Platypleurini to date but also the earliest fossil record of the subfamily Cicadinae. The age of *E. messelensis* gen. et sp. nov. from the Eocene Messel has been determined to be approximately 47.22 million years, which is significantly older than the divergence time of about 39 million years proposed for the tribe Platypleurini and its sister groups in molecular studies e.g.,^50,81^. Additionally, this age predates the estimated most recent common ancestor (MRCA) of the Platypleurini crown group, which was dated to about 25 million years ago (with a 95% confidence interval spanning 30 to 20 million years)^50^. Our new discovery suggests that previous estimates for the origin time of the Platypleurini crown group and the divergence time with its sister groups may have been underestimated. The discovery of *E. messelensis* gen. et sp. nov. indicates that the separation time between the Platypleurini and its sister groups was no later than 47 Ma. This new evidence is significant for revising the timing of internal nodes in the phylogenetic analysis of the Cicadidae.

### Implications for the paleoenvironment and geographic distribution of the tribe platypleurini

The Messel site, recognized as a significant Eocene Lagerstätte, hosts a diverse array of fossils, encompassing vertebrates, invertebrates (notably insects), and plants among others e.g.,^82–84^. The mean annual temperature at Eocene Messel have been reconstructed based on analyses of plant fossils and the δO values of vertebrate tooth apatite^53,60^, these reconstructions estimate a mean annual temperature of between 18 °C and 22 °C. Annual precipitation ranging from 803 to 2,540 mm, and an average relative humidity of 73–77%, aligning with the characteristics of a modern subtropical humid climate^60^. Studies of the plant assemblages from Messel suggest that its vegetation corresponds to that of a paratropical forest^85^. Therefore, ecological reconstructions of the Messel site indicate that *E. messelensis* gen. et sp. nov. inhabited a relatively humid forest environment.

The extant Platypleurini are predominantly found in sub-Saharan Africa, the eastern Arabian Peninsula, South Asia, East Asia, and Southeast Asia. Their habitats are mainly characterized by climatic zones such as the humid subtropical climate (Cwa and Cfa), subtropical highland climate, tropical monsoon climate (Am), tropical savanna climate (Aw), and tropical rainforest climate (Af) (Fig. 2b). These regions typically experience substantial annual or seasonal precipitation and relatively high temperatures^51^, with the predominant vegetation being forest environments.

In addition, some extant Platypleurini can also be found in more arid environments, such as the lineages inhabiting the arid to semi-arid climates (Fig. 2b, Bwh and Bsh) of southern Africa, and *Platypleura arabica*, which is found in the arid climate of the eastern Arabian Peninsula. Some species are capable of living in cooler environments compared to subtropical climates, such as *Planopleura kaempferi*, which inhabits areas with hot-summer humid continental climates (Fig. 2b, Dwa, Dfa) in northern East Asia.

According to Price et al. (2019)^50^, extant Platypleurini can be divided into three major lineages. The earliest lineage is primarily distributed in Central Africa and Madagascar. The second major lineage is found across Asia, and the third lineage is mainly distributed in southern Africa, with a smaller number of species inhabiting Central Africa. The earliest-diverging African and Asian lineages predominantly inhabit forest environments with high precipitation in subtropical and tropical regions. In contrast, the majority of the third, more recently diverged lineage is adapted to the arid and semi-arid environments of southern Africa, with a few species found in the forests of Central Africa. The habitat of the Messel cicada closely resembles that of the more basal lineages of extant Platypleurini, further supporting the hypothesis that the most recent common ancestor of extant Platypleurini likely inhabited forest environments with abundant precipitation and temperature.

The paleolatitude of the Eocene Messel fossil site is estimated to be around 46° to 47° N^60^, higher than the current latitudinal range of extant Platypleurini. During this period, higher global temperatures facilitated a humid subtropical climate at these higher latitudes, suggesting that Platypleurini, along with potentially other cicada groups, may have historically occupied a much broader geographical range, extending into mid- to high-latitude regions. However, the marked global cooling from the Eocene to the Oligocene, followed by a transition into ‘coolhouse’ and ‘icehouse’ climate periods^86^, likely led to increased aridification and cooling of these regions. Such environmental changes presumably prompted a southward shift in the habitat range of Platypleurini. This contraction in range likely played a pivotal role in shaping their current distribution, now confined mostly to low- to mid-latitude areas, and may have driven the adaptation of some species within the Platypleurini to more arid and cooler climates.

Despite the current low diversity and abundance of Cicadidae species in Europe and in Germany in particular, fossil records from the Eocene, Oligocene, and Pliocene indicate that Germany was once a significant region of high cicada diversity, as evidenced by the rich fossil findings (Table 1; Fig. 1). Furthermore, extensive cicada fossils from the Miocene have been documented in various European locations, including the Czech Republic, France, Croatia, and Switzerland (Table 1; Fig. 1). In addition, there are still many Cicadidae fossils from Messel waiting to be described, including those mentioned in earlier literature^87^ and further excavations in recent years. These data collectively underscore that the cicadas had a widespread distribution across the European continent during the Cenozoic.

**Fig. 10:**
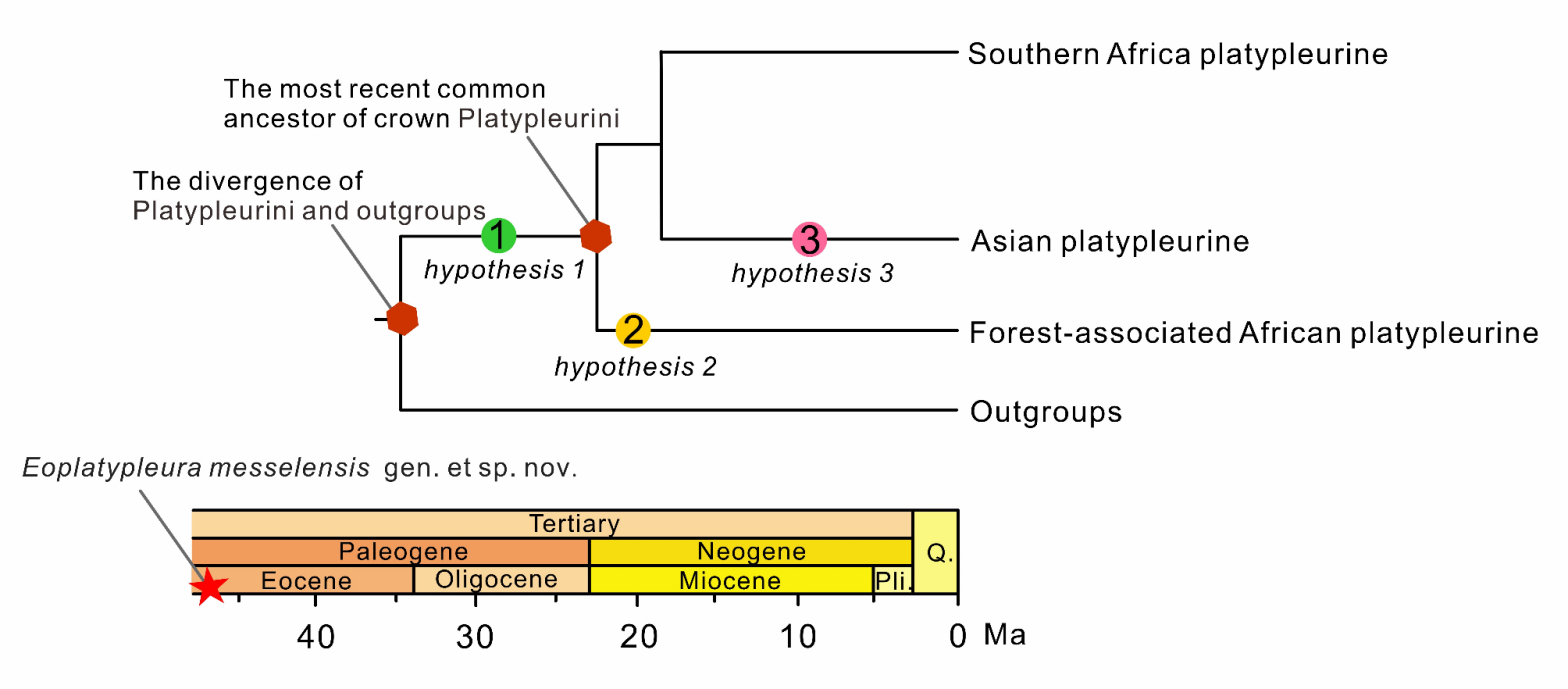
Simplified diagram of molecular phylogenetic results of the cicada tribe Platipleurini, modified from Price et al., 2019^50^, showing the phylogenetic placement of *Eoplatypleura messelensis* gen. et sp. nov., based on hypotheses 1, 2, and 3.

### Implications and hypotheses on the paleogeographic distribution and dispersal of the tribe platypleurini

Currently, the tribe Platypleurini is primarily distributed across sub-Saharan Africa, South Asia, East Asia, and Southeast Asia, as depicted in Fig. 3, which maps the global distribution based on records from the Global Biodiversity Information Facility (GBIF, https://www.gbif.org) and iNaturalist (https://www.inaturalist.org/). We excluded scattered records from Europe and North America from the map, as they were neither formally documented nor indicative of native populations. Possibly, such records are related to specimens introduced through international transport of commercial goods. According to Price et al. (2019)^50^, the modern Platypleurini likely originated in Africa, and the populations currently found on the Eurasian continent are thought to have dispersed from Africa to Asia following the continental collision between Africa and Eurasia approximately 30–25 million years ago. The cicada fossils from the Platypleurini tribe at the Messel site may hold significant paleogeographic implications, which depend on their contingent upon their phylogenetic positions (Fig. 10), as discussed below.

#### Hypothesis 1: *E. messelensis* gen. et sp. nov. belongs to the stem group of the tribe Platypleurini

If this hypothesis holds true, *E. messelensis* gen. et sp. nov. might represent a stem group member that existed on the Eurasian continent before the emergence of the extant Platypleurini. According to the ancestral state reconstruction by Price et al. (2019)^50^, the sister groups to Platypleurini are predominantly found across the Asian continent. Therefore, the divergence between the Platypleurini and their sister groups likely occurred on the Eurasian continent, with the divergence timing confirmed to be no later than 47 Ma. *E. messelensis* gen. et sp. nov. exhibits morphological features akin to those of extant Platypleurini, indicating that the fundamental morphological traits of the tribe had already evolved prior to the emergence of their crown group.

#### Hypothesis 2: *E. messelensis* gen. et sp. nov. belongs to the lineage of African forest Platypleurini

Price et al. (2019)^50^ conducted a phylogenetic analysis and exploratory reconstruction of ancestral areas, revealing that the basal clade of the Platypleurini comprises members from Madagascar and equatorial Africa inhabiting forest environments. They propose that the Platypleurini originated in equatorial Africa and were adapted to forested habitats. If *E. messelensis* gen. et sp. nov. belongs to the part of this African forest clade, it would suggest that the origin of the crown group of Platypleurini dates back significantly earlier than previously estimated, with a minimum age no later than 47 Ma. Furthermore, it would imply that the divergence of the African forest clade also occurred no later than 47 Ma. In this scenario, *E. messelensis* gen. et sp. nov. might represent a separate dispersal event out of Africa, distinct from the dispersal event leading to the extant Asian Platypleurini.

#### Hypothesis 3: *E. messelensis* gen. et sp. nov. belongs to the lineage of the extant Asian Platypleurini

According to the phylogenetic analysis by Price et al. (2019)^50^, the extant Asian tribe Platypleurini forms a monophyletic group, representing a single dispersal event out of Africa. This event occurred approximately 20 Ma ago, subsequent to the collision between the African and Eurasian plates. If *E. messelensis* gen. et sp. nov. belongs to part of this clade, it would suggest that the origin of the extant Asian Platypleurini dates back to at least 47 Ma, considerably earlier than the 20 Ma estimated by molecular clock analyses. This would further imply that the dispersal of Platypleurini out of Africa occurred as a single event.

Based on molecular studies suggesting an African origin for the modern Platypleurini^50^ and our fossils, hypothesis 1 proposes the dispersal of stem group Platypleurini from Eurasia to Africa before the origin of the crown group of Platypleurini (approximately 30− 20 million years ago). Both hypotheses 2 and 3 suggest the dispersal of crown group Platypleurini from Africa to Eurasia prior to 47 Ma. Ezcurra & Agnolín (2012)^88^ pointed out that the faunal and floral assemblages of Europe during the Paleocene and Eocene exhibit phylogenetic similarities with those of Africa, South America, and Australia, and proposed a biogeographic connection between Europe and Africa during this period. Smith et al. (2016)^89^ demonstrated that early Eocene vertebrate fossils from the western Indian subcontinent share similarities with those of Europe and Africa, suggesting biogeographic connection between these regions via the Indian subcontinent. These biogeographic findings would support the hypotheses that Platypleurini dispersed from Africa to Europe before 47 Ma (hypotheses 2 and 3) or they dispersed from Europe to Africa before 25 Ma (hypothesis 1), based on the estimated origin time of the crown group Platypleurini from Price et al. (2019).

Our fossil evidence suggests that the Platypleurini and its outgroups might have diverged at or by 47 Ma which is significantly earlier than the time estimated by molecular clock analyses of Price et al. (2019)^50^. Prior to our study, no fossils of the tribe Platypleurini had been discovered. Price et al. (2019)^50^ relied on only two fossil calibrations: *Cryptotympana miocenica* Zhang & Zhang (16.0–11.6 Ma)^33^ and *Meimuna protopalifera* Fujiyama (23.6–16 Ma)^36^. A key reason for the discrepancies in origin time between fossil records and molecular clock estimates might be that the limited number of available fossils introduced biases in time estimates. In the future, incorporating additional fossil evidence into studies is expected to improve the accuracy of temporal calibrations and molecular clock analyses, providing a clearer understanding of the evolutionary history and divergence times of Platypleurini.

## Concluding summary

**Fig. 11:**
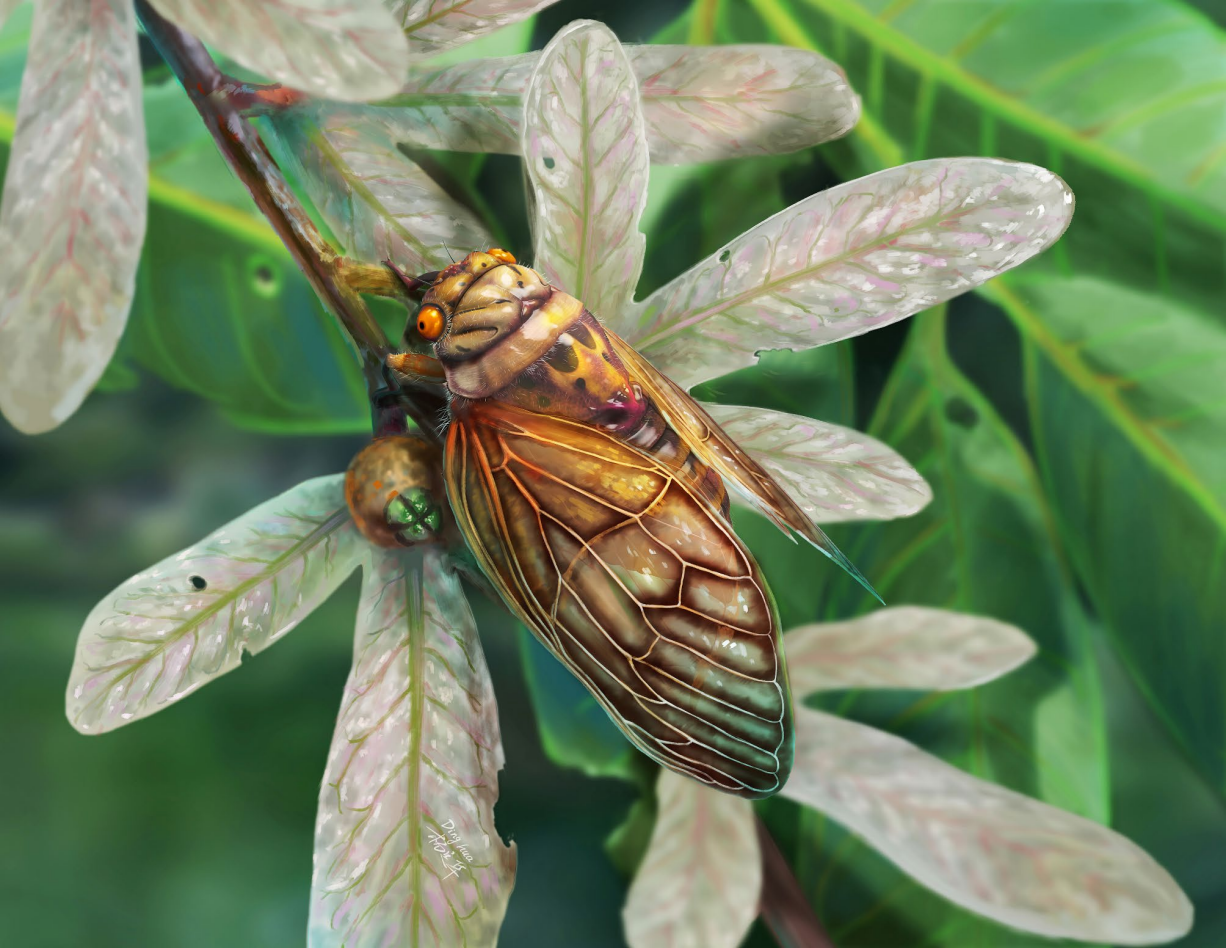
Reconstruction of *Eoplatypleura messelensis* gen. et sp. nov. from the Eocene Messel Pit, by Dinghua Yang.

This study reports the new genus and species, *Eoplatypleura messelensis* (artistic reconstruction in Fig. 11), and discusses several hypotheses concerning the paleogeographic distribution and dispersal of the tribe Platypleurini. With the discovery of additional fossils and advances in molecular research, these hypotheses are expected to be further explored, refined, and validated in future studies. Our research underscores the critical role of fossil records in elucidating the origins, geographic distribution, responses to climate and evolutionary processes of modern insects, as well as in informing our understanding of the diversity and dispersal patterns of contemporary taxa.

## Data Availability

All data generated or analyzed during this study are included or sources are cited in this published article. The fossil specimens and datasets used and/or analysed during the current study available from the corresponding authors on reasonable request.
